# Reconsidering self‐deprecation as a communication practice

**DOI:** 10.1111/bjso.12329

**Published:** 2019-05-18

**Authors:** Susan A. Speer

**Affiliations:** ^1^ Division of Psychology and Mental Health School of Health Sciences The University of Manchester UK

**Keywords:** conversation analysis, identity, meta‐talk, preference organization, self‐criticism, self‐esteem

## Abstract

‘Self‐deprecation’ (SD) is widely understood within social psychology and popular culture as a form of self‐talk that reflects a cognitive state, such as low self‐esteem or negative self‐regard. However, most research on SD suffers theoretical and methodological problems that fail to account for how its cognitive and linguistic aspects can be reconciled. We know little about SD as it occurs in interactional settings. Utilizing a conversation analytic (CA) perspective that brackets cognitive explanations for linguistic phenomena, this paper draws on more than 100 hours of transcribed recordings of interactions from diverse settings to systematically examine the form and function of a common class of SD: critical comments by a speaker on their current talk or actions (self‐deprecatory meta‐comments; SDMCs). Analyses demonstrate that SDMCs are used in environments of possible or actual interactional trouble, and manage this trouble in different sequential positions. The paper shows that SDs can be treated as a communication practice. Rigorous analysis of SDMCs can enrich understanding of the construction of ‘identities’ in talk. It advances a CA understanding of the ascription of social actions, and the preference for self‐criticism over criticism by others. Findings suggest that widespread advice to self‐deprecate less may be invalid.

## Background

‘Self‐deprecation’ (SD) or ‘negative self‐evaluation’ (Owens, 1994) is widely understood within psychology and popular culture as a form of ‘self‐talk’ that reflects a cognitive state or personality dimension, such as low self‐esteem or negative self‐regard (Owens, 1993, 1994; Owens, Stryker, & Goodman, 2006; Rhodewalt & Agustsdottir, 1986; Rosenberg, Schooler, Schoenbach, & Rosenberg, 1995; Sciangula & Morry, 2009). Commonly treated as any statement that conveys a negative self‐evaluation, typical examples of SD from the literature include explicit statements containing negative assessments such as ‘at times I think I am no good at all’ and ‘I feel useless at times’, and less obvious constructions that imply a deficit such as ‘I wish I could have more respect for myself’ (Owens *et al*., 2006: 183).

Psychologists argue that a self‐deprecatory ‘tendency to denigrate’ or ‘disparage’ oneself (Owens *et al*., 2006), is associated with depression and anxiety (Kopala‐Sibley, Klein, Perlman, & Kotov, 2017; Luyten *et al*., 2007; Owens, 1994) and is a form of ‘self‐sabotage’ that can leave others believing the disparaging things we say about ourselves (Breuning, 2016; Chandler, 2017; O'Malley, 2017). Regarded by some as a gendered trait that is used more by women than men (O'Malley, 2017; see also Greengross & Miller, 2008), commentators suggest that overuse of self‐deprecatory statements can damage relationships and ‘leave the receiver feeling burdened to prop up the self‐deprecator’ (Chandler, 2017). Instead of self‐deprecating, we are advised to use self‐talk positively to help manage our fears (through the use of affirmations, for example) (Weintraub, 2015).

A number of studies investigate the ‘presentation management’ aspects of SDs, showing that individuals may ‘vary the positivity of their self‐presentations’ in different contexts (Schlenker & Leary, 1982: 90; see also Beer, Chester, & Hughes, 2013; Tice, Butler, Muraven, & Stillwell, 1995). For example, it is argued that we may self‐deprecate to convey modesty (Blickle, Diekmann, Schneider, Kalthöfer, & Summers, 2012; Robinson, Johnson, & Shields, 1995), adjusting our self‐presentational style to produce ‘optimal audience reactions’ (Schlenker & Leary, 1982: 102). Some suggest that SD is ‘the cornerstone of British humour’ (Chandler, 2017; O'Malley, 2017), whose use is increasing, particularly amongst social media users (Parkinson, 2016). SD humour may be used by high‐status individuals as ‘a way of transiently faking inferior personally traits’ so as to appear agreeable and win support (Greengross & Miller, 2008: 404; Stewart, 2011), or by politicians to ‘increase their favorability ratings’ (Baumgartner, Morris, & Coleman, 2015). SDs may be used as an ‘appeasement strategy’ by the outperformer of the outperformed (Zell & Exline, 2010). Finally, some researchers suggest that self‐criticism and self‐enhancement can coexist (Preuss & Alicke, 2017) and that individuals can enhance their present selves by disparaging their past selves (Wilson & Ross, 2001).

These studies appear to suggest that SDs may be used as a linguistic device to achieve beneficial outcomes, and hence acknowledge the importance of social context in shaping their meaning. However, most research on SD suffers theoretical and methodological problems that limit its usefulness: Theoretically, it fails to account for how the cognitive and linguistic aspects of SDs can be reconciled. For example, it is not clear how SDs can be indicative of a cognitive state such as low self‐esteem, with negative consequences for the individual and their relationships, on the one hand, and used skilfully as a linguistic device for positive self‐presentational purposes, on the other.

Methodologically, most research on SD relies on tickbox questionnaire measures such as Owens *et al*. (2006: 183) adaptation of the 10‐item Rosenberg self‐esteem scale (Rosenberg, 1986), which reduces SD to decontextualized, global self‐esteem statements. Alternatively, it is based on evidence collected under experimental, laboratory conditions and from other artificial situations where participants are invited to produce SDs by researchers ‘on demand’ (e.g., Beer *et al*., 2013; Sciangula & Morry, 2009; Zell & Exline, 2010). Therefore, the emphasis to date has been on the production of SDs as a largely individualized, solitary endeavour. We know little about how SDs are interactionally organized and responded to, or about the form and function of SDs as they occur in settings that are not specifically set up for the purposes of eliciting negative self‐talk.

A handful of studies influenced by linguistics and conversation analysis (CA) have analysed naturally occurring language use to shed light on some of these issues. Pomerantz (1984) examined SDs, which she defined as ‘self‐critical assessments’ (Pomerantz, 1984: 78), as part of her study on agreeing and disagreeing with assessments. She found that the interactionally ‘preferred’ response to an initial assessment is an agreeing second assessment (e.g., *A:* ‘It's a beautiful day out isn't it?’, B: ‘Yeh it's just gorgeous’). By contrast, the preferred response to a self‐deprecation is disagreement: (e.g., A: ‘I'm so dumb I don't even know it’, B: ‘No, you're not dumb’). She argued that overtly agreeing with a self‐deprecation is a *dispreferred* action because it is tantamount to disaligning with the speaker and endorsing the prior criticism (1984, p. 81).

Pomerantz's research significantly extends what we know about responses to SDs. However, she analysed them in the context of ‘unspecified, friendly conversation’, which impacts the preference structures she identified (Kotthoff, 1993: 213) and leaves open questions about the broader applicability of her findings. Analysis of SDs in other settings suggests a different preference structure: Kotthoff (1993: 196) argues that in therapeutic settings, the preferred response to an SD is not disagreement. Rather, the SD is treated by the therapist as a matter to explore. Similarly, Lazaraton's (1997) analyses of interviews for admission to an ‘English as a Second Language’ class show that students’ SDs of their English language competence (e.g., ‘I feel my oral English is not good’) are usually met with silence by the interviewer rather than the preferred, disagreeing response highlighted by Pomerantz: To disagree with their SD would be tantamount to rejection from the course, since it would indicate that they do not, in fact, need help with their English language.

Where Pomerantz and colleagues focused primarily on *responses* to SDs, others have considered their *interactional functions*: Researchers have demonstrated that SDs are a resource for a range of social actions (e.g., making complaints, attributing or countering blame) that display interactional competence and involve both identity and moral work by the speaker (Burch, 2017). They can be used by speakers to ‘level with each other and become of “one mind”’ (Kim, 2014), as a resource for building ‘affiliation and solidarity’ (Kim, 2015), or to save the ‘face’ of the speaker or their recipient (Yu, 2013).

A few studies point to the trouble management function of SDs: Whitehead (2013; see also Matwick & Matwick, 2017) shows how self‐deprecating racial categorizations can be used to ‘inoculate’ the speaker against sanction for any offence caused, while Hendry, Wiggins, and Anderson (2015) suggest that group deprecations can be used following a period of trouble, to build group cohesiveness. Finally, Childs and Walsh (2017) show how self‐deprecating self‐references (e.g., ‘I'm going deaf that's all’) can be used by police officers to personalize their investigative interviews with children who have reported being the victim of a sexual offence.

These studies advance our understanding of the interactional functions of SDs in the discrete settings analysed: Data sources include mundane conversations in Japanese (Burch, 2017), task‐based conversations in Korean and Japanese (Kim, 2014), Korean telephone conversations (Kim, 2015), informal interactions amongst university students (Yu, 2013), South African radio call‐in shows (Whitehead, 2013), the talk of celebrity chefs (Matwick & Matwick, 2017), student problem‐based learning tutorials (Hendry *et al*., 2015), and investigative police interviews (Childs & Walsh, 2017). However, we do not know whether particular classes of SD have generic interactional functions across contexts, or anything about the sequential environments or slots in which SDs occur. Therefore, for a more complete understanding of SDs we need to conduct a rigorous analysis of the interactional work they do and where they do it across a range of settings.

This paper fills this knowledge gap by using CA to systematically examine the composition, sequential position, and interactional function of a common class of SD, namely self‐deprecatory ‘meta‐comments’ (SDMCs; Schiffrin, 1980). As a subclass of SDs, SDMCs are also ‘self‐critical assessments’ (Pomerantz, 1984; Schegloff, 2007) that perform a self‐critical *action,* directly or indirectly expressed. However, the distinguishing feature of SDMCs is that the object they critically assess is *the speaker's current talk or behaviour*. Thus, utterances like ‘I sound ageist’, ‘I have gone off the point’, and ‘you're getting an earful of this’ were all included as instances of SDMCs in the data‐trawl for this paper. Non‐lexical or embodied actions that display a self‐deprecatory stance (e.g., face palm, ‘duh!’) were not included as examples of SD, but represent a potential avenue for future work.

Self‐deprecatory meta‐comments are a methodologically ‘ideal’ class of SD to analyse, since the object being criticized (the speaker's current talk or actions) is endogenous to the interaction evidenced within the transcript, and hence open to full analytic scrutiny. This contrasts with other classes of SD (e.g., ‘I'm rubbish at maths’) where the object of the SD (evidence for the speaker's maths ability) may be exogenous to the interaction analysed. Hence, analyses can help us to understand precisely which features of an interaction are oriented towards and treated by members as particular kinds of social actions (i.e., they display a member’s understanding of what counts as ‘being unfair’, ‘sounding ageist’, or ‘drifting off’).

## Method

Data consist of a corpus of more than 100 hours of transcribed recordings of interactions from diverse settings:

1. British and American audio and video recordings of ordinary, peer group, and family interactions, recorded in the 1960s and 1970s and used widely by conversation analysts.
2. The ‘Holt’ corpus of British telephone calls between family members, friends, and acquaintances recorded in the 1980s and used widely by conversation analysts.
3. 194 routine psychiatric consultations (made up of 156 audio recordings and 38 video recordings) collected from a UK National Health Service Gender Identity Clinic between 2004 and 2008 (Speer & Green, 2008). The sample comprised recordings made by four psychiatrists with 182 consecutive consenting patients. Ethical approval was granted by the NHS Central Office of Research Ethics Committees.
4. 21 semi‐structured research interviews collected between 2013 and 2014 by two female researchers with men from the United Kingdom who had been treated for prostate cancer. Five of the men were accompanied, at their request, by their female partner who also gave their consent and actively participated in the interview. Ethical approval was granted by The University of Manchester Research Ethics Committee.
5. Two publicly broadcast television programmes recorded by the author on an *ad hoc* basis upon hearing an SDMC.

Using data from a diverse range of ordinary and institutional settings increases the broad applicability of the findings and allows us to identify, for the first time, both systematic, setting‐specific variations in the interactional practices identified and their generic or ‘context‐free’ features (Drew, 2003; Speer, 2012a).

The entire corpus was systematically searched to identify 43 instances of SDMC. Instances were transcribed using Jefferson's (2004) conventions for CA which represent talk in greater detail than verbatim transcription, so that its subtle nuances are captured and can be analysed (see Table 1). In all but Extract 4, where it has been historical practice to retain identifying features in the data (see Schegloff, 2007), identifying details were replaced with pseudonyms.

**Table 1:** Transcription symbols (adapted from Jefferson, 2004)

Aspects of the relative placement/timing of utterances		
=	Equals sign	Immediate latching of successive talk
(0.8)	Time in parentheses	The length of a silence, in tenths of a second
(.)	Period in parentheses	A silence that is less than a tenth of a second
[overlap]	Square brackets	Mark the onset and end of overlapping talk
Aspects of speech delivery		
.	Period	Closing, usually falling intonation
,	Comma	Continuing, slightly upward intonation
?	Question mark	Rising intonation
Underline	Underlining	Talk that is emphasized by the speaker
Rea::lly	Colon(s)	Elongation or stretch of the prior sound
c:	Underlining preceding colon	When letters preceding colons are underlined, the pitch rises on the letter and the overall contour is ‘up‐to‐down’
:	Underlined colon	Rising pitch on the colon in an overall ‘down‐to‐up’ contour
!	Exclamation mark	Animated tone
‐	Hyphen/dash	A sharp cut‐off of the prior word or sound
↑	Upward arrow	Precedes a marked rise in pitch
↓	Downward arrow	Precedes a marked fall in pitch
<	‘Greater than’ sign	Talk that is ‘jump‐started’.
>faster<	‘Lesser than’ & ‘greater than’ signs	Enclose speeded up or compressed talk
<slower>	‘Greater than’ & ‘ lesser than’ signs	Enclose slower or elongated talk
LOUD	Upper case	Talk that is noticeably louder than that surrounding it
ºquiet º	Degree signs	Enclose talk that is noticeably quieter than that surrounding it.
huh/hah/heh/hih/hoh		Various types of laughter token
(h)	‘h’ in parentheses	Audible aspirations within speech (e.g., laughter particles)
.hhh	A dot before an h or series of h's	An inbreath (number of h's indicates length)
hhh	An h or series of h's	An outbreath/breathiness (number of h's indicates length)
$ or £	Dollar or pound sign	Smile voice
*	Asterisk	Squeaky vocal delivery
( )	Empty single parentheses	Non‐transcribable segment of talk
(talk)	Word(s) in single parentheses	Transcriber's possible hearing
(it)/(at)	A slash separating word(s) in single parentheses	Two alternative transcriber hearings
((laughs))	Word(s) in double parentheses	Transcriber comments or description of a sound

Instances were analysed using CA (see Sidnell, 2013, for an overview). CA has been used to great effect to consider in interactional terms what is traditionally conceived as a psychological or cognitive matter (Mikesell *et al*., 2017; Potter & Edwards, 2013), including the production of identities in talk (Antaki, Barnes, & Leudar, 2005; Antaki & Widdicombe, 1998; Widdicombe, 2017). CA does not deny the existence of thoughts and feelings. Rather, it treats talk as action rather than a tool to transmit what is inside our heads. By bracketing cognitive explanations for interactional phenomena, and considering the interactional organization of ‘identity talk’, CA offers an alternative framework for understanding SDs as a communication practice.

A distinguishing feature of CA is its focus on both the *composition* of individual turns as well as their *position* in a sequence. Sequences matter because they are the mechanism through which courses of social action (including SDs) are implemented and responded to, and through which mutual understanding is arrived at and displayed (Schegloff, 2007).

Analyses proceeded as follows: Taking each instance in turn, transcripts were read alongside the original sound or video file with a view to identifying recurrent patterns in the composition, interactional function, and sequential position of SDMCs. Instances were analysed in detail by considering the words, phrases, and grammatical design of turns containing SDMCs, the actions they formed part of, and their relative position in a sequence (i.e., analyses considered what came before and after the SDMC and the responses (if any) that they engendered). Next, analyses identified examples of the sequential environments or ‘slots’ in which SDMCs occur. Instances were selected for inclusion in the paper that show important patterns and variations within each sequence type.

## Results

Self‐deprecatory meta‐comments are commonly used by speakers in environments of possible or actual interactional trouble, where there are or may be communication difficulties or breakdowns in mutual alignment. SDMCs work to manage or pre‐empt and deflect this trouble in different sequential positions or ‘slots’ relative to the (potentially) problematic action sequence. Analyses are divided into sections that reflect these different slots (the total number of instances of each type is given in brackets):

1. SDMCs that *precede* a potentially problematic action sequence (n=6)
2. SDMCs that address a potentially problematic *action‐in‐progress* (n=12)
3. SDMCs that *close* potentially problematic action sequences (n=5)
4. SDMCs that *expand* potentially problematic action sequences (n=19)

The majority of SDMCs in the corpus work in a pre‐emptive fashion to name, manage, or deflect interactional troubles that have not been made explicit by the recipient. In section five, by contrast, I consider a deviant case of an SDMC that is used *post hoc*, as a ‘last resort’ move to manage the explicit, repeated criticism of the recipient, and transition out of a problematic action sequence (*n* = 1). I end by considering the implications of these analyses for a social psychological understanding of negative self‐descriptions, and a CA approach to action formation.

### 1. SDMCs that precede a potentially problematic action sequence

In the first subset of the data, the SDMC *precedes* talk that the speaker casts as potentially troublesome or offensive to the recipient. Examples of such ‘forward‐looking’ preliminaries include: ‘I'm going to sound really silly’ (Psychiatric consultation 128) and ‘I'm gonna come over as ageist, I'm gonna come across as a terrible person actually’ (Interview 21). Extract 1 is a typical example. The analysis will draw out some key features of SDMCs positioned in this slot, before demonstrating, in the following sections, some sequential variations on the features identified.

#### Extract 1

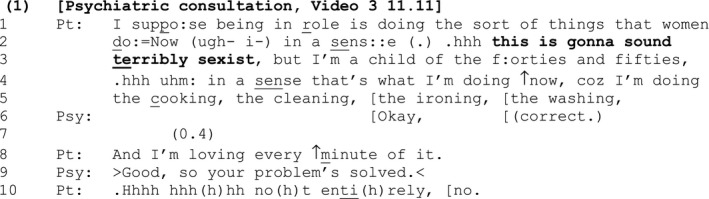

Extract 1 is from a psychiatric consultation between a natal male who wishes to undergo medical treatment to become a woman and a male psychiatrist (the patient is referred to using pronouns that reflect the gender she identifies with). The psychiatrist has asked the patient to explain a prior statement where she indicated that she needed to spend more time living ‘in role’ rather than ‘just dressing’ as a woman. The SDMC ‘this is gonna sound terribly sexist’ (lines 2–3) is delivered as part of the patient's explanation.

Designed to be heard as a maximally built, negative assessment (it is ‘terribly’ sexist, not just ‘sexist’ (Pomerantz, 1986)), the SDMC is inserted parenthetically into, and temporarily halts the progressivity of the turn, in order to inform the recipient ‘how to listen to the turn in progress’ (Mazeland, 2007: 1816). Bracketed by the repeat of ‘in a sense’ (lines 2 and 4), the parenthetical material serves to announce a forthcoming offence on the speaker's part that is evidenced by the patient's claim that she is ‘loving every minute of’ performing the stereotypically female tasks of ‘the cooking, the cleaning, the ironing, the washing’ (lines 4–5 and 8).

When considered in individualized, psychological terms, the SDMC might be interpreted as evidence for the patient's self‐critical personality, or lack of confidence in what she is about to say. However, when its design and placement is considered from an interactional perspective that brackets cognitive explanations, other possible, ‘action‐based’ explanations emerge:

The SDMC ‘this is gonna sound terribly sexist’ (lines 2–3) works rather like a disclaimer (e.g., ‘*I'm not racist but…*’, ‘*I don't mean to be rude but…’*; Hewitt & Stokes, 1975; Overstreet & Yule, 2001) to inoculate the speaker against criticism for the offence that it announces (cf. Whitehead, 2013). However, instead of denying a problematic identity (as with a disclaimer), the SDMC takes ownership of, and acknowledges a potentially problematic identity.

Interestingly, the parenthetical material also contains an account: ‘but I'm a child of the forties and fifties’ (line 3). Thus, while the SDMC claims *interactional* responsibility for *saying something* that may sound sexist, the account mitigates the patient's *personal* responsibility for that potential sexism. In other words, by suggesting that she is a victim of history, the patient positions herself as not wholly accountable for the sexism that she claims.

By using the SDMC, the patient is able to get her view ‘on record’ at the same time as demonstrating to the psychiatrist that she is a reflexive analytic being who is attentive to the possibility that her forthcoming talk may be vulnerable to criticism (and hence she is not unthinkingly celebrating gender stereotypes). Indeed, it is perhaps no coincidence that in this ‘gatekeeping’ setting where the patient is required to live as a woman in order to gain access to treatment (Speer & Parsons, 2006), the position she marks as troublesome (‘loving’ behaving in a stereotypically female way) aligns with a strongly ‘pro treatment’ position – in that she is and should be a woman.

Interestingly, the psychiatrist responds (line 9) to the patient's positive assessment (line 8) by commenting on its implications for the patient's ‘problem’ (that she does not actually have a problem that requires treatment if she is happy as she is): (‘>Good, so your problem's solved.<’). He does not respond to the SDMC itself, by commenting on whether or not she sounded sexist. Indeed, unlike the SDs that Pomerantz (1984) considered, since this SDMC is delivered parenthetically as a metacommunicative *announcement* regarding how the *forthcoming* talk may sound, it is not designed for an agreeing/disagreeing response.

In sum, the SDMC in Extract 1 is used in a pre‐emptive fashion to head off offence or criticism, by marking forthcoming talk as problematic. In doing so, it makes a potentially problematic claim ‘safely sayable’ and helps the speaker to maintain a positive identity for herself at precisely the moment where such an identity may be most at risk.

In the next two instances, the speaker halts the progressivity of the ongoing sequence to deliver an ‘apology plus SDMC’ that addresses a potentially problematic *action‐in‐progress*.

### 2. SDMCs that address a potentially problematic action‐in‐progress

Lesley has called Robbie to discuss some photographs. However, Robbie uses the opportunity afforded by Lesley's call to report troubles with her role as the teacher of a class that Lesley appears to have also taught. Robbie seeks Lesley's advice regarding how to manage a child called Gabriel (lines 1–3 and 9).

#### Extract 2

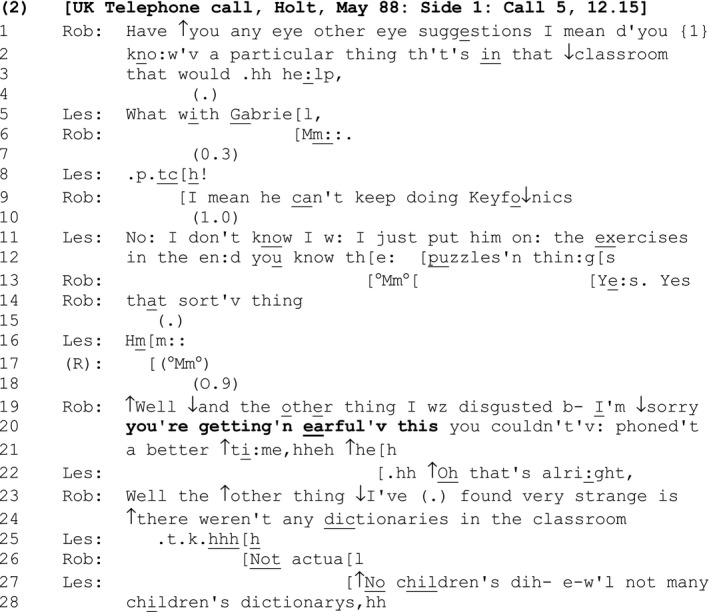

Following Lesley's advice, a lull materializes where neither party takes the floor (line 18). Robbie initiates a further troubles report (line 19), prefaced with the discourse particle ‘well’, which functions as an alert that the turn in progress ‘will privilege its speaker's perspectives, interests or project relative to the expectations for action established in the prior turn or sequence’ (Heritage, 2015: 88). Indeed, having indicated that she is about to report something that ‘disgusted’ her, which may alert Leslie to a forthcoming complaint, Robbie temporarily halts the progressivity of her turn in progress, in order to initiate a parenthetical sequence (lines 19–20) that metacommunicatively informs Lesley how she should hear the ongoing talk.

Bracketed by a repeat of ‘Well… the other thing’ (lines 19 and 23), this parenthetical contains an apology for some offence, ‘I’m ↓sorry’, an SDMC that names the offence, ‘you're getting'n earful'v this’, and, as before, an account that mitigates personal responsibility for that offence: ‘you couldn't'v: phoned't a better ↑ti:me,hheh ↑he[h’ (lines 19–20). This account jokingly presents Lesley as partly responsible for any suffering she may be experiencing by virtue of the unfortunate timing of her call (Cirillo, Colón de Carvajal, & Ticca, 2016; Heritage & Raymond, 2016).

‘You're getting ‘n earful'v this’ (line 20) constitutes an SDMC because it evaluates Robbie's current actions (in this case what she is saying to Leslie – ‘of this’) in negative terms (‘getting an earful’), with the preceding apology treating ‘giving someone an earful’, as an offence. Unlike the SDMC in Extract 1, this SDMC is designed to be heard as a negative assessment of the speaker's *current* (‘you're getting…’) and not *forthcoming* (‘this is gonna sound…’) actions. Built as an announcement, the SDMC reflexively marks and manages an ongoing offence on the speaker's part that may index a negative identity: in this case as someone who has misappropriated someone else's call to repeatedly offload their troubles (giving the recipient an ‘earful’, is colloquial for ‘going on too much’).

By using the ‘apology plus SDMC’ format, Robbie demonstrates to Lesley that she is reflexively attuned to the possibility that her ongoing actions may be causing offence, and seeks to be absolved of guilt for that offence, thus pre‐emptively inoculating herself against criticism for it.

Interestingly, although the laughter tokens appended to the account that follows the SDMC treat is as non‐serious (‘you couldn't'v: phoned't a better ↑ti:me,hheh ↑he[h’, lines 19–20) and thereby invites laughter (line 21; Glenn, 2003), Lesley does not join in. This reflects a pattern highlighted by Jefferson (1984) who showed that while a troubles teller may laugh, the recipient, ever attentive to the ongoing sequence *as* a troubles report, rarely does so. Instead, Lesley absolves Robbie of guilt for any offence (line 22). By doing so, she gives Robbie a licence to continue her troubles report, while leaving open the possibility that Robbie has indeed given her an ‘earful’. This may account, in part, for why, when Robbie does resume the sequence, she also *downgrades* her complaint from ‘the other thing I wz disgusted b’ to the rather more measured: ‘the ↑other thing ↓I've (.) found very strange’ (line 23).

Extract 3, from a research interview, shares some similar features.

#### Extract 3

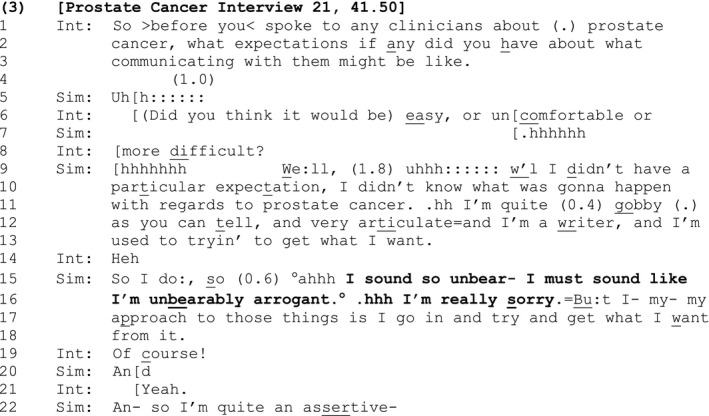

The interviewer asks Simon what expectations (if any) he had regarding his communication with the doctor about his prostate cancer (lines 1–3). Right away, there is evidence that Simon's response may not be straightforward: The lengthy delays (lines 4–5), provision of candidate answers by the interviewer (lines 6 and 8), Simon's sighing, turn initial ‘well’ (line 9; Heritage, 2015), and further delays (line 9) suggest the interviewer's question about his expectations may be inapposite. Indeed, Simon eventually notes that he ‘didn't have a particular expectation’ (lines 9–10).

In formulating the upshot of his response (So I do:, line 15), Simon initiates a parenthetical sequence (bracketed by ‘So’, lines 15 and 22) that temporarily halts the progressivity of the turn in progress in order to metacommunicatively deal with any offence, before resuming the potentially problematic course of action (line 22). Like the previous instance, the parenthetical material contains the SDMC, ‘I sound so unbear‐ I must sound like I'm unbearably arrogant’ (lines 15–16); an apology, ‘I'm really sorry’ (line 16); and an account that apparently mitigates personal responsibility for any offence, ‘Bu:t I‐ my‐ my approach to those things is I go in and try and get what I want from it’ (lines 16–18).

As before, the SDMC forms an announcement that is designed to be heard as a maximal negative assessment of the speaker's actions (‘unbearably arrogant’ rather than just ‘arrogant’). It reflexively assumes the critical perspective of the other in order to orient towards, and inoculate the speaker against, some current (‘I sound’/’I must sound’), interactional offence (sounding ‘unbearably arrogant’). Indeed, the SD is positioned following a list of ‘self‐promoting’ descriptions that might suggest ‘arrogance’ on Simon's part: He has described himself as a ‘very articulate’ writer who is ‘used to tryin’ to get what I want’ (lines 12–13). As conversation analysts have shown, praising oneself is a dispreferred action that is vulnerable to criticism or accusations of ‘bragging’ (Pomerantz, 1978) and speakers commonly employ various strategies to minimize or back away from it (Speer, 2012b).

Interestingly, in this instance the SDMC is itself repaired from ‘I sound’ to ‘I must sound’, whereby Simon shifts from a position of epistemic certainty regarding how he comes across to others, to the downgraded, comparatively less certain ‘I must sound like I'm unbearably arrogant’ (lines 15–16). By building the SDMC in this way, Simon appears to seek the participation of the interviewer, and her confirmation or disconfirmation of his negative self‐assessment. In the notable absence of such participation, or indeed of any (audible) response from the interviewer, Simon attends to the possibility that he may have *actually* caused offence by apologizing (line 16). Interestingly, he does not leave a slot for a response following his apology, but immediately appends the account that mitigates personal responsibility for any offence caused (lines 16–18).

It is only at this point that the interviewer responds with ‘of course’ (line 19), which reinforces the self‐evident correctness of Simon's approach (cf. Stivers, 2011), while leaving open or equivocal the matter of whether or not he sounded ‘arrogant’.

So far, analyses have considered the form, function and sequential position of SDMCs that are positioned *before* (section one) or *during* (section two) some potential interactional trouble or offence. In section three, I examine two SDMCs that form part of, and facilitate, *sequence closings*. These SDMCs retrospectively cast the sequences they close as troublesome at the same time as pre‐empting, and inoculating the speaker against, criticism for any offence caused.

### 3. SDMCs that close potentially problematic action sequences

In Extract 4, the SDMC forms part of a turn that announces the closure of a sequence that the speaker casts as problematic. Stan has telephoned his sister, Joyce, to ask her advice about where he can buy a hat and sandals:

#### Extract 4

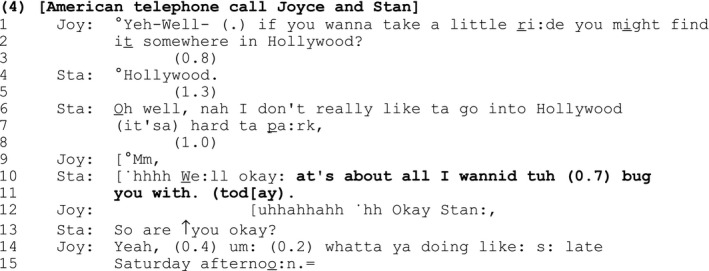

Stan has repeatedly pursued but then resisted Joyce's advice. Stan's final act of resistance – ‘nah I don't really like ta go into Hollywood (it'sa) hard ta pa:rk’ (lines 6–7) – amounts to a rejection of Joyce's advice that is not well received – note Joyce's delayed, minimal response to that rejection (lines 8–9).

The SDMC ‘at's about all I wannid tuh (0.7) bug you with. (tod[ay)’ (lines 10–11) comes at precisely the point where Stan is most at risk of being accused of ‘bugging’ (a colloquial term for ‘annoying’ or ‘bothering’) Joyce. Characterizing his prior actions in negative terms, the SDMC forms part of an announcement of the ending of the current activity and closure of the sequence.

Stan appears to use the SDMC at this point in order to transition in a ‘light‐hearted’ manner out of a lengthy sequence that has become troublesome. By taking the critical perspective of the other to name and own his possible offence, Stan reflexively demonstrates his orientation to his prior actions as potentially troublesome at the same time as deflecting criticism for them.

Joyce's laughter (line 12) demonstrates that she declines to take Stan's SDMC seriously, while her compliance token (‘okay’, line 12) aligns with Stan's move to close the sequence (Schegloff, 2002: 261).

Extract 5 comes towards the end of a British Professional cyclist's acceptance speech at the 2011 BBC Sports Personality of the Year Awards.

#### Extract 5

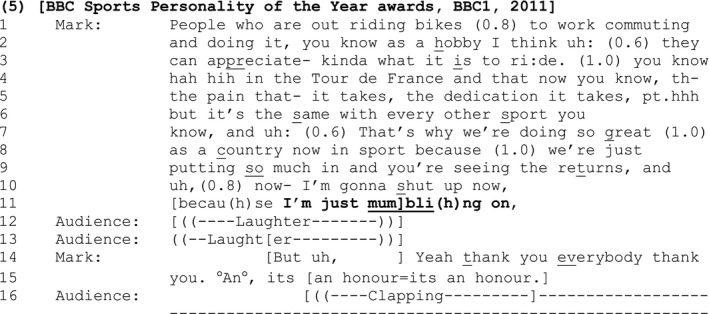

Mark's SDMC ‘I'm just mumbl(h)ing on’ (line 11) forms part of an account for the closing of the current activity, signalled by Mark's announcement that he is going to ‘shut up now’ (line 10). The speech so far has lasted for two minutes and 33 seconds. Generally, long acceptance speeches are vulnerable to criticism (e.g., Kate Winslet's 2011 Emmy Awards acceptance speech was labelled ‘rambling’ by critics (McQueen, 2011)). Hence, this SDMC comes at precisely the point in the speech where Mark may be criticized for ‘mumbling on’.

As before, by deploying the SDMC at this point, Mark shows that he is a reflexive analytic being, attuned, and willing to draw attention to his interactional deficiencies for the benefit of diffusing tension and maintaining good relations with his audience. Indeed, interaction external, ‘exogenous evidence’ (Speer, 2017), suggests that Mark's speech was well received in the popular media – being later characterized as ‘heartfelt and ‘rather sweet’ (White, 2011).

Of course, in this particular setting, the speaker is delivering a monologue to an overhearing audience who cannot criticize directly (beyond, possibly, shaking their heads or resorting to heckling). Therefore Mark claims what might normatively be the critical perspective of the overhearing audience in order to self‐criticize. By pre‐emptively announcing an offence, Mark deflects criticism for his actions. In doing so, he transitions out of what might objectively be regarded as a rather formal or serious sequence, with mutual affiliation evidenced by all parties. Thus, by inflecting the turn that contains the SDMC with laughter (line 11), it is designed to be heard as non‐serious and invite reciprocal laughter – which it gets (line 12 onwards, Glenn, 2003). As before, this response aligns with the course of action initiated by the turn containing the SDMC (positive sequence closure), but leaves open the question of whether or not the SDMC was valid.

Extract 4, Extract 5 show that when a sequence that is potentially troublesome and which threatens mutual affiliation, reaches possible closure, participants can address that trouble and close the sequence using an SDMC. One way that speakers manage problematic sequences that have already reached possible closure is to *expand* them using SDMCs that ‘check out’ the possibility that prior comments may have caused offence.

### 4. SDMCs that expand potentially problematic action sequences

In Extract 6, the patient is challenged by the psychiatrist to explain why she is convinced that undergoing medical treatment for gender dysphoria is a ‘good thing to be doing’ at this point in her life, when she has not done anything about it during the previous four years.

#### Extract 6

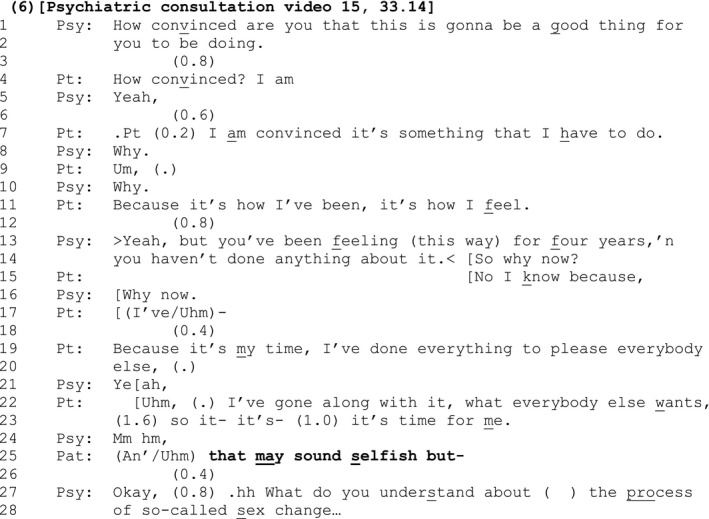

The patient sets out her ‘bottom line’ explanation for why she wants treatment now (lines 19–20 and 22–23). At line 23, her response is complete grammatically, intonationally, and in action terms. Indeed, the ‘upshot’ of her position (‘it's time for me’, line 23), reiterates the view she first expressed at line 19 (‘it's my time’). However, following the psychiatrist's response (line 24), which acts as a continuer that prompts for more, she expands her response with the trailed off SDMC ‘that may sound selfish but‐’ (line 25).

By metacommunicatively evaluating, in negative terms, the view she has just expressed in her prior turn, the patient shows herself to be orienting to that view as one where she may have ‘gone too far’ in expressing her sense of entitlement, thus exhibiting negative features of her identity (that she is selfish). By voicing such a potential criticism as an SDMC, she pre‐empts and deflects accusations of selfishness at precisely the point in the interaction where her comments may be most at risk of such a criticism.

Like Extract 3, the SDMC in this instance is built in an epistemically weak fashion since it seeks the psychiatrist's confirmation or disconfirmation of its veracity (‘may sound’ selfish, line 25). However, the psychiatrist sequentially deletes the SDMC with a closing implicative ‘Okay’ (line 27, Schegloff, 2007), before initiating a new action sequence (line 27).

The following instance, taken from a research interview with a man with prostate cancer, is one of the few examples in the corpus where the recipient of the SDMC appears to explicitly reject it in the preferred manner first described by Pomerantz (1984):

#### Extract 7

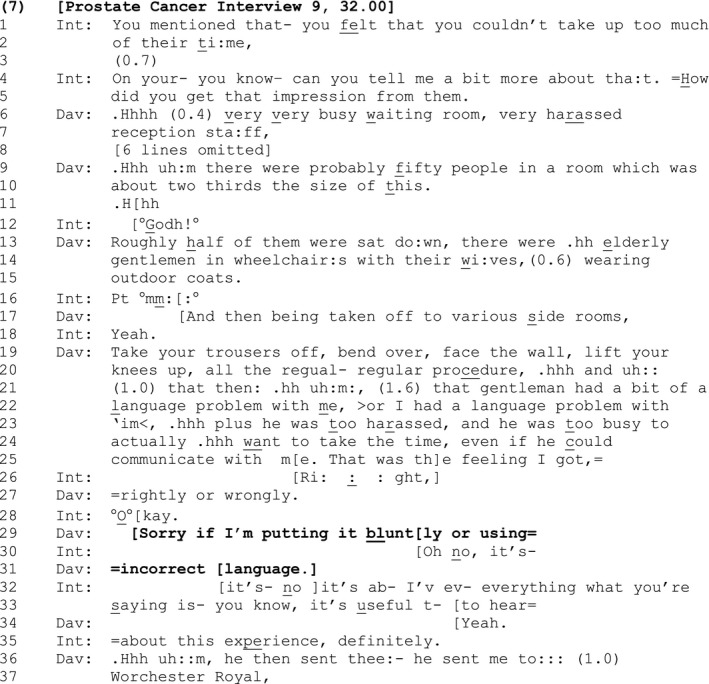

During a lengthy critical commentary David makes some hearably racist remarks regarding the language abilities of one of the doctors who ‘had a bit of a language problem with me’ (lines 21–22). Perhaps orienting to the vulnerability of his remarks to accusations of racism, he puts forward a potential alternative explanation in which responsibility for the language problem lay with him (>or I had a language problem with ‘im< (lines 22–23). In what appears to be a further attempt to deflect any suggestion that he is locating the source of the problem in the individual doctor or his language abilities, David locates the ‘bottom line’ source of his difficulty with features of the doctor's workload, which meant that he was too ‘harassed’ and ‘busy’ to want to communicate with him ‘even if he could communicate with me’ (lines 23–25). Although this suggests the doctor's communication abilities were, for David, irrelevant to his overall view of him, this comment nonetheless reinstates David's earlier suggestion that the doctor had poor communication skills, and hence remains vulnerable to accusations of prejudice.

A common response to a complaint is empathy of some kind, particularly in the context of a research interview where the interviewer may be attentive to rapport building (Lavin & Maynard, 2001). However, empathizing with David's remarks could be problematic, since it would leave the interviewer vulnerable to suggestions that she has endorsed racism within the course of her (audio‐recorded) work. The interviewer‘s use of 'right’ (line 26) navigates a relatively neutral path through these issues by claiming epistemic progression in response to David's point, while withholding affiliation or empathy (Gardner, 2007).

David recompletes his turn in overlap with the interviewer's response, acknowledging that the view he reports may be morally problematic (lines 25 and 27). This is receipted by the interviewer with a closing implicative ‘Okay’ (line 28). It is at this point, following the interviewer's minimal and non‐empathic response to his complaint and the apparent closure of the sequence, that David expands it by attending to the potential trouble or offence he may have caused: The apology and SDMC, ‘[Sorry if I'm putting it blunt[ly or using= =incorrect [language.]’ (lines 29 and 31), is seemingly used here as a euphemism for ‘politically incorrect’.

Notice that once again, David delivers these SDMCs in an apologetic, epistemically weak fashion with respect to any offence caused: ‘If I'm putting’ seeks reassurance from the interviewer regarding the veracity or correctness of his negative assessments and, if offence has been caused, absolution from guilt. David thereby uses the SDMC to bring to the interactional surface, and work to manage and deflect criticism for, a potentially racist action.

Interestingly, unlike previous instances, where the recipients of the SDMC do not comment directly on its validity (as in Extracts 1–5), or else sequentially delete it (as in Extract 6), the interviewer in this sequence initially disagrees with the SD in a ‘preferred’ pattern first highlighted by Pomerantz (1984): The interviewer dismisses the suggestion that David's views sounded blunt or that he used incorrect language (lines 30 and 32) and notes instead that his views on his experience are ‘useful’ to hear (i.e., they have utility as data for an interview on this topic), while avoiding affiliating with them. In this way the interviewer manages the delicate task of providing reassurance, maintaining rapport, and valuing the interviewee's views *as* views, without condoning prejudice. Having received the interviewer's assurances, David resumes view‐giving (line 36).

In sections 1‐4, the SDMCs made relevant and managed interactional troubles in a pre‐emptive fashion, such that those troubles are not made explicit by the recipient. Extract 8 represents a rare example that demonstrates what happens when a speaker commits an interactional offence and is directly criticized and teased by their recipient for that offence. In this case, the speaker uses the SDMC in a *responsive* fashion, as a ‘last resort’ move to transition out of the troublesome action sequence. I want to suggest that this instance may provide evidence for an interactional preference for self‐criticism over criticism by others.

### 5. SDMCs used *post hoc* to manage explicit criticism

#### Extract 8

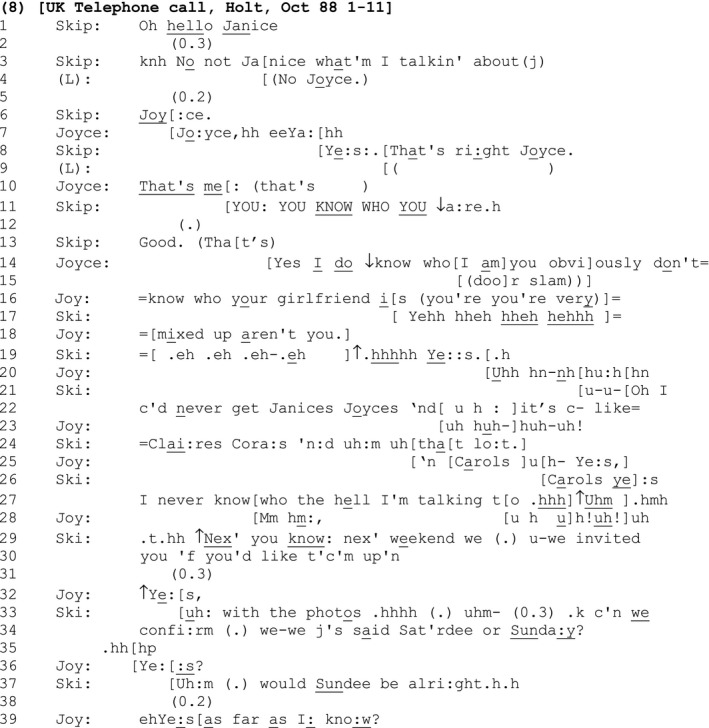

Skip has called his friend Joyce, but misidentifies her by calling her ‘Janice’ (line 1). Such a misidentification is a highly accountable action, and indeed the progressivity of the call is temporarily held in abeyance until it is addressed.

After a short silence in the slot where one might expect the recipient of the call to confirm it is them and return the greeting (line 2), Skip notices, and initiates repair on his error, chastises himself using the reflexive meta‐commentary ‘what'm I talkin’ about’ (line 3), before providing the correct identification, in overlap with Joyce's correction (lines 6 and 7). They both reconfirm the correctness of that identification (lines 8 and 10).

Skip attempts to make light of his interactional *faux pas* by highlighting the obvious fact that Joyce clearly knows who she is (with the self‐mocking inference that *he* clearly does not) (line 11). However, instead of ‘letting it go’ there, Joyce's delayed response (line 14) expands the sequence apparently in order to repeatedly re‐aggravate the offence and hold Skip directly accountable for his error: She begins with a marked full repeat of what Skip has said, teasingly mocking him with ‘Yes I do ↓know who[I am]you obvi]ously don't know who your girlfriend i[s’ and ‘you're very mixed up aren't you’ (lines 14, 16, and 18) (Drew, 1987).

Skip responds to this, first with laughter (lines 17 and 19), and then by agreeing with the negative assessment that he is very mixed up (line 19). Joyce joins in with the laughter (line 20), before Skip issues the first of two SDMCs: ‘Oh I c'd never get Janices Joyces ‘nd[u h :]it's c‐ like=Clai:res Cora:s ‘n:d uh:m uh[tha[t lo:t.]’ (lines 21, 22, and 24). This SDMC announcement serves to mitigate Skip's faux pas as a routine trouble he has with certain names (‘I c'd never get…’, lines 21–22) rather than a one‐off personal failing or something particularly forgettable about Joyce. Joyce does not let Skip ‘off the hook’ but agrees with and embellishes his SDMC, expanding it on his behalf (line 25) (cf. Pomerantz, 1984).

Skip then issues the further SDMC announcement: ‘I never know[who the hell I'm talking t[o .hhh]↑Uhm].hmh’ (line 27). Notice that this SDMC upgrades the prior self‐deprecation which had suggested that Skip had a past problem with specific names, to one that suggests a current, all‐encompassing problem with person identification (Edwards, 1994). This second SDMC works as a ‘last resort’ move on Skip's part to take ownership of the offence and transition out of the problematic sequence. Indeed, since he builds it as a maximal self‐criticism, Joyce has no further comeback and Skip initiates a new action sequence (line 29).

## Discussion

This paper has systematically analysed the form, function and sequential organization of SDMCs across a substantial corpus of data from diverse, ordinary and institutional settings. I argued that SDMCs are a methodologically ideal class of SD to analyse, since the object being criticized (the speaker's current talk) is endogenous to the interaction and transcript under investigation, and hence open to full analytic scrutiny.

Results demonstrate that SDMCs may be a generic conversational device that works in similar ways across ordinary and institutional settings (Drew, 2003; Speer, 2012a): SDMCs are commonly used by speakers in environments of possible or actual interactional trouble, to orient towards and manage the possibility that they have committed, or may be about to commit, some interactional ‘offence’. These potential troubles or offences are constructed by participants as follows: sounding sexist, giving someone an ‘earful’, sounding arrogant, mumbling on, ‘bugging’ one's recipient, sounding selfish, speaking bluntly or using incorrect language, and never knowing who one is talking to. All of these offences are ones where the speaker may risk negative identity attributions being made about them.

Analyses demonstrate that SDMCs do ‘identity work’ by managing, pre‐empting and deflecting this trouble, in different sequential positions or ‘slots’ relative to the (potentially) problematic action sequence (i.e., they are positioned before, during, at the closure or expansion of, the sequence). The majority of SDMCs analysed here named and managed interactional troubles that were implicit within the interaction: The recipient of the SDMC did not explicitly draw attention to these troubles, or criticize the SDMC speaker. Extract 8, by contrast, represents a deviant case that illustrates what happens when the offending party is directly and repeatedly criticized for their interactional offence (misidentifying the person they have telephoned). In this case, instead of using the SDMC to inoculate the speaker against criticism, the offending party uses the SDMC in a responsive fashion, as a last resort move to manage the criticism *post hoc*, and transition out of the problematic action sequence.

To the extent that in all of the cases analysed here, the recipient of the SDMC does not directly agree with it (i.e., none of the recipients agree with the claim that the speaker sounds sexist, selfish, arrogant, blunt, is mumbling on, bugging them, or giving them an earful, and so on), and their response helps to advance the interactional project of the SDMC speaker (the continuation or closing of the sequence), they can be considered ‘successful’. As Pomerantz has shown, to endorse or agree with an SD would be considered a *dispreferred* action because to do so would be tantamount to criticizing the speaker (1984: 81; for more on preference organization, see Pillet‐Shore, 2017; Pomerantz & Heritage, 2013). Therefore, one of the virtues of SDMCs, and a reason why they may be such a powerful interactional resource across settings, is that the SDMC speaker can exploit the preference structure of SDs highlighted by Pomerantz in order to get ‘on record’ and make ‘safely sayable’ precisely the kinds of claims that may be risky or vulnerable to criticism. In order to draw attention to some interactional trouble or offence *after* an SDMC has been issued in respect of that trouble or offence, the recipient must perform a dispreferred action, with the associated risks to social solidarity (Lindstrom & Sorjonen, 2013) and progressivity of the sequence. Such a move would also be somewhat redundant: If speaker A declares that what they are about to say may be rude or arrogant, it would make little sense for speaker B to then criticize A for being rude or arrogant: it would be a case of ‘I told you so’. The SDMC works to deflect such criticism by pre‐emptively ‘disarming’ the recipient to detoxify any possible trouble.

The sheer frequency of cases where SDMCs are used in this *pre‐emptive* fashion as a ‘first resort’ move to inoculate the speaker against criticism (as in Extract 1, Extract 2, Extract 3, Extract 4, Extract 5, Extract 6, Extract 7), rather than in a *responsive* fashion as a ‘last resort’ move to manage actual criticism (as in Extract 8), may provide evidence for an interactional preference for self‐criticism or self‐sanctioning over criticism and sanctioning by others. In other words, it may be better to reflexively ‘notice’, take responsibility for, and declare one's own interactional shortcomings rather than have them pointed out or ‘announced’ by others (cf. Pillet‐Shore, 2017; Speer, 2012b). Indeed, the disruption to the progressivity of the sequence that ensues in Extract 8 after the speaker's misidentification of his recipient, and the comparatively delayed SDMC in response to repeated criticism for that offence, provides further evidence to support this possibility. Importantly, this preference for noticing one's own faults over announcement by others, reverses the preference structure at play when orienting to normatively *positive* features of identity (e.g., a new haircut or changed appearance), where there is a preference for noticing by others over announcement by self (Schegloff, 2007: 82; Speer, 2012b). Further research is needed to explore the context sensitive nature of this preference and the interactional circumstances where self‐deprecations may be positive for social solidarity.

As I have noted, in many respects, SDMCs act rather like disclaimers (e.g., I'm not racist/sexist but…, I don't mean to be rude but…’) which are also bound up in the interactional management of identity (Hewitt & Stokes, 1975; Overstreet & Yule, 2001). However, although SDMCs and disclaimers both work to inoculate the speaker against *negative* identity attributions, SDMCs do so by *claiming* negative identity attributes, while disclaimers *deny* such attributes. In many respects, however, disclaimers have become so familiar as to be caricatures of themselves, vulnerable to being called out by others *as* strategic in nature (e.g., the disclaimer ‘I don't mean to be rude but…’ is defined as ‘a precursor to being intentionally rude’ (Urban Dictionary, 2011)). By contrast, the use of self‐deprecations is thought to be increasing (Parkinson, 2016, and cf. Schegloff, 2007: 225, who states ‘self‐deprecation is not all that common an occurrence’): In a culture where we are routinely urged to self‐deprecate less (and use positive affirmations more), SDs may be a particularly powerful interactional tool precisely so as to appear *non‐strategic*.

This is not to suggest that SDMCs never reflect cognitions or a negative view of self when used by individuals who score poorly on questionnaire measures of self‐esteem. Rather, I hope to have demonstrated that a substantial proportion of the SDMCs in the data analysed here, like positive self‐descriptions (Speer, 2012b) can be explained, in large part, in interactional, rather than cognitive, terms, as a *communication practice*.

This interactional approach to identity presents a challenge to much social psychological work that treats self‐descriptions as a reflection of an internal state or personality dimension, and to the self‐help genre that promotes the use of positive affirmations (and avoidance of negative self‐talk) as a route to feeling good about ourselves and maintaining positive relationships. Indeed, findings suggest that psychologists may gain a richer understanding of negative self‐descriptions, and the range of mechanisms that are at play when identities are made relevant in talk, by considering the interactional organization of instances of ‘identity talk’ (Antaki & Widdicombe, 1998; Speer, 2012b; Widdicombe, 2017).

I want to end by reflecting on the implications of these analyses for a CA understanding of *action formation*: The data presented here demonstrate a members’ explicit orientation towards, and labelling of, the *kinds* of talk that are recognizable as particular kinds of actions (sounding sexist, giving someone an ‘earful’, sounding arrogant, mumbling on, ‘bugging’ one's recipient, sounding selfish, and speaking bluntly or using ‘incorrect’ language). As Levinson (2013: 128) notes, analyses of such moments, where ‘action ascription is overtly under scrutiny by participants’, can significantly advance our understanding of ‘what counts’ as a particular action type and the process of assigning an action to a turn (Levinson, 2013: 104; see also Speer, 2017). Analysis of SDMCs can also make a potentially fruitful contribution to the advancement of this CA project.

## Data archiving statement

For ethical reasons, data cannot be publicly shared, but detailed transcripts are included in the article so that analyses can be independently validated.
